# Frontotemporal Dementias: A Review

**DOI:** 10.1186/1744-859X-6-15

**Published:** 2007-06-12

**Authors:** Natalie D Weder, Rehan Aziz, Kirsten Wilkins, Rajesh R Tampi

**Affiliations:** 1 Department of Psychiatry, Yale University School of Medicine, New Haven, CT, USA

## Abstract

Dementia is a clinical state characterized by loss of function in multiple cognitive domains. It is a costly disease in terms of both personal suffering and economic loss. Frontotemporal dementia (FTD) is the term now preferred over Picks disease to describe the spectrum of non-Alzheimers dementias characterized by focal atrophy of the frontal and anterior temporal regions of the brain. The prevalence of FTD is considerable, though specific figures vary among different studies. It occurs usually in an age range of 35–75 and it is more common in individuals with a positive family history of dementia. The risk factors associated with this disorder include head injury and family history of FTD. Although there is some controversy regarding the further syndromatic subdivision of the different types of FTD, the three major clinical presentations of FTD include: 1) a frontal or behavioral variant (FvFTD), 2) a temporal, aphasic variant, also called Semantic dementia (SD), and 3) a progressive aphasia (PA). These different variants differ in their clinical presentation, cognitive deficits, and affected brain regions. Patients with FTD should have a neuropsychiatric assessment, neuropsychological testing and neuroimaging studies to confirm and clarify the diagnosis. Treatment for this entity consists of behavioral and pharmacological approaches. Medications such as serotonin reuptake inhibitors, antipsychotics, mood stabilizer and other novel treatments have been used in FTD with different rates of success. Further research should be directed at understanding and developing new diagnostic and therapeutic modalities to improve the patients' prognosis and quality of life.

## Background

Dementia is a clinical state characterized by the loss of function in multiple cognitive domains. It is a costly disease in terms of both personal suffering and economic loss. Hence, an understanding of its prevalence, risk factors, prompt diagnosis methods and potential interventions is critical [1]. In terms of frontotemporal dementia, it has been more than 110 years since Arnold Pick described the first of a series of cases, which separated focal atrophies from what was at that time called senile atrophy. The eponym "Pick's disease" was suggested by a pupil of Pick who had thought that the frontal and temporal lobes, seen as phylogenetically younger, were more vulnerable to degenerative disease. Later, pathologists began restricting the use of the term 'Pick's' disease to refer only to the pathologic finding of Pick's bodies. This created the impression that Pick's disease was rare and difficult to diagnose [2]. Over time, when physicians began encountering patients with frontal degeneration and personality change (disinhibition), they would often diagnose it as Pick's disease. However, subsequently, these patients were rarely found to have actual Pick's bodies [3].

The term is now used to describe what has been discovered to be a host of related neurodegenerative conditions. These conditions are characterized by disturbances in behavior or language. Unfortunately, there is considerable confusion in the literature regarding FTD because authors have used different nomenclature to describe similar clinical entities and because symptoms of FTD are related to the anatomical areas affected rather than to precise neuropathological entities. Further complicating matters is that FTD, refers to both the overall name of this group of diseases and to the clinical subgroup mainly affecting the frontal lobes, i.e. frontal-variant FTD [3]. Despite this overlap, the division of FTD into three main subgroups has been widely accepted. These include the frontal-variant or behavioral-variant (fvFTD also just called FTD); progressive nonfluent aphasia (PNFA); and semantic dementia (SD). The motor syndromes of corticobasal degeneration (CBD); progressive supranuclear palsy (PSP); and motor neuron disease may also be associated with features of FTD and its pathology. Because of this association, they have been included as part of the same spectrum of disorders [4]. Of note, other authors have suggested the terms Pick complex [5] or dysexecutive syndrome [6] be incorporated instead.

FTD has an heterogenous pathology. The pathological profile is characterized by gliosis, neuronal loss, and superficial spongiform degeneration in the frontal and/or temporal cortexes. Ballooned neurons, i.e. Pick cells, occur with variable frequency in all subtypes [7]. Further, the fact that tau-inclusions have been confirmed in FTD, CBD and PSP have made some authors indicate that the pathology of FTD should be divided into tau-positive and tau-negative variations and that the clinical picture only differs, again, because of the affected brain regions [8,9].

This article reviews recent literature on FTD's epidemiology, clinical presentation, diagnosis, neuropathology, and treatments.

### 2. Epidemiology

Two recent studies have addressed the prevalence of FTD. Ratnavalli et al., reported a prevalence of 15 per 100,000 in a population between 45 and 64 years of age [10], while Rosso et al., [11] who studied the prevalence of FTD in the Netherlands, reported an overall prevalence of FTD of 1.1 in 100,000 with a maximum prevalence of 9.4 per 100,000 for ages 60–69. Overall, FTD is estimated to account for 20% of cases of degenerative dementia with presenile onset [12]. Post-mortem investigations have reported a relative frequency of FTD of 3–10% [9].

Frontotemporal dementia usually affects people in the age range of 35–75 years. Among these patients, about 20–40% have a positive family history for FTD [13]. Two recent studies have reported that the incidence rates for FTD (new cases per 100,000 person-years) were 2.2 for ages 40 to 49 years, 3.3 for ages 50 to 59 years, and 8.9 for ages 60 to 69 years. In comparison, the corresponding rates for Alzheimer disease were 0.0, 3.3, and 88.9 years respectively [14,15]. Although the age of onset tends to be younger than in patients with AD, it doesn't seem to vary between familial and sporadic cases [16]. The median age of onset of FTD is about 58 with 22% of the patients having an age of onset after age 65.7.

In a recent study by Hodges et al., median survival from symptom onset was found to be 6 +/- 1.1 years for FTD and 3 +/- 0.4 years for FTD-MND. The median survival for the entire group was 3.0 years from the time of diagnosis, and 75% were dead within 6.0 years. This short survival was attributable partly to delayed diagnosis; on average, 3.0 years elapsed between symptom onset and diagnosis. However, one of the group's most striking findings was that the institutionalization occurred on an average of only 1.0 year after the diagnosis was made [17]. In further support of this finding, Roberson et al reported that FTD progresses faster than Alzheimer's disease (AD). Survival from presentation was estimated to be 5.7 years, in comparison to 11.7 years in patients with AD. Some of the factors associated with a decreased survival in FTD included the presence of Amyotrophic Lateral Sclerosis (ALS), specific degeneration of frontal-subcortical circuits, and tau-negative cases. They also found that patients with semantic dementia (SD) had significantly longer survival compared to other subsets of FTD [18].

### 3. Risk Factors

Few studies have reported on specific risk factors for FTD. Recognized risk factors include family history of FTD and a personal history of head trauma. A case-control study that included 80 cases of sporadic FTD reported a significant association between FTD and head trauma. There was also a positive association between thyroid disease and the risk of FTD. Specifically, thyroid disease was associated with a 2.5 times increased risk of frontotemporal dementia. This was not statistically significant (p = 0.09), though, owing to limited power of this study[19].

### 4. Clinical presentation

FTD results in behavioral, cognitive and neurological changes. These three major clinical presentations are described below. Generally, in terms of behavioral alterations, patients often tend to lack appropriate basic and social emotions. Some patients with FTD present with disinhibition and overactivity, while others show apathy and blunted affect [20]. Some behavioral abnormalities seen in patients with FTD have been compared to those presented by patients with antisocial personality disorder. Functional imaging studies have shown abnormalities in individuals with acquired sociopathy that involve the same areas affected in FTD. These include the anterior temporal lobes and ventromedial frontal and orbitofrontal cortex [21]. It has also been suggested that these patients suffer from "moral agnosia", which could be related to an inability to differentiate right from wrong or from the loss of the capacity to reason [22].

Patients with FTD show marked deficiencies in executive functioning and working memory [20]. Other frequently encountered cognitive abnormalities include deficits in attention, poor abstraction, difficulty shifting mental set, and perseveration [12]. Interestingly, spatial skills seem to remain unaffected in such patients [20].

Neurological signs are usually absent early in the disease, although patients may display primitive reflexes. With disease progression, patients develop parkinsonian signs of akinesia and rigidity, which can be prominent. They may also have repetitive motor behaviors and muscular rigidity [20]. A minority of patients develops neurological signs consistent with motor neuron disease [23].

The symptoms of FTD reflect the distribution of the pathological changes rather than the precise histological subtype. The degree of frontal vs. temporal pathology can account for additional variability in the presenting symptoms of FTD [24]. Complicating matters further, patients may initially present with symptoms consistent with one particular FTD syndrome but then progress to a different FTD subtype [25].

The three major clinical presentations of FTD include: 1) a frontal or behavioral variant (FvFTD), 2) a temporal, aphasic variant, also called Semantic dementia (SD), and 3) a progressive aphasia (PA). Table 1 summarizes the differences in clinical presentation, cognitive deficits, and affected brain regions of the three variants.

**Table 1:** Common clinical presentations of the various types of frontotemporal dementia.

	Common Initial	Behavioral Symptoms	Cognitive Symptoms	Commonly Affected
	Presentation			Brain Region
FvFTD^a^	Personality	Occur Early:	Executive dysfunction	Frontal/prefrontal
	change	Disinhibition	Impaired working memory	cortex
		Impulsivity	Perseveration	Anterior temporal
		Stereotypies	Attentional deficits	cortex
		Apathy		
		Hyperorality		
SD^b^	Language	Occur Early or Late:	Fluent dysphasia	Middle and inferior
	abnormality	Emotional distance	Impaired semantic memory	temporal neocortex
		Interpersonal coldness	Preserved autobiographical	
			and working memory	
PA^c^	Language	Occur Late:	Non-fluent/expressive	Left perisylvian
	abnormality	May include any of the	dysphasia	cortex
		above		

^a ^= Frontal variant frontotemporal dementia; ^b ^= Semantic dementia; ^c ^= Progressive aphasia

#### A. Frontal Variant FTD (FvFTD)

FvFTD is characterized by the insidious onset of personality changes, behavioral abnormalities and poor insight. The division of frontal lobe function into three separate areas (orbitobasal, medial, and dorsolateral) offers a way to understand the clinical presentation of FvFTD. Orbitobasal involvment leads to some of the most common symptoms encountered in this disorder. These include disinhibition, poor impulse control, antisocial behavior, and stereotypical behaviors. Examples of stereotypical, or ritualized behaviors, include insisting on eating the same food at exactly the same time daily, cleaning the house in precisely the same order, or simple repetitive behaviors such as foot-tapping. Ritualized acts may also include the use of a "catch-phrase" and a change in food preference [26]. A patient's decline in social conduct can include breaches of interpersonal etiquette and tactlessness. Verbally inappropriate sexual comments and gestures are common [27].

Apathy is correlated with the severity of medial frontal-anterior cingulate involvement [26]. Dietary changes are frequent and typically take the form of overeating, i.e. hyperorality, with a preference for sweet foods [26]. Patients also exhibit emotional blunting. Speech output is attenuated and mutism eventually develops. Echolalia and perseveration may be present.

The most common cognitive deficit in FvFTD is an impairment of executive function or working memory [27], which is indicative of frontal and prefrontal cortex involvement. Other frequently encountered cognitive abnormalities include attentional deficits, poor abstraction, difficulty shifting mental set, and perseverative tendencies [12]. Deficits in planning, organization, and other aspects of executive function become universal as the disease progresses, and this reflects the involvement of the dorsolateral prefrontal cortex [26].

Within the clinical subtypes of FvFTD, there is marked heterogeneity of clinical presentations, often as a result of differential involvement of brain regions. Some patients are disinhibited, fatuous, purposelessly overactive, easily distracted, socially inappropriate, and lacking in concern. At the other extreme, others are bland, apathetic, inert, lacking volition, mentally rigid, and perseverative [12]. Social behavior has been shown to be more disrupted in patients with predominantly right-hemisphere pathology [23]. Moreover, Mc Murtray., [24] demonstrated that patients with frontal FTD showed hypoactivity and apathy, whereas patients with temporal FTD showed hypomania-like behavior. Decreased insight was associated with right frontal hypoperfusion, while decreased hygiene and grooming with left frontal hypoperfusion. Patients with left hemisphere FTD had early speech and language difficulty but greater normal behavior, whereas patients with right hemisphere FTD had normal speech and language but more frequent inappropriate behavior [24].

#### B. Semantic Dementia (SD)

Temporal FTD, also known as semantic dementia (SD), is associated with bilateral atrophy of the middle and inferior temporal neocortex [20]. The most common initial presentation in these patients is an abnormality of language, which includes loss of memory for words or a loss of word meaning [27]. Patients with SD are often unaware of their difficulties with comprehension. Speech is fluent, but patients tend to use substitute phrases such as "thing" or "that" [27]. Patients lose the ability to name and understand words and to recognize the significance of faces, objects and other sensory stimuli. They also show deficits on non-verbal tasks using visual, auditory, and other modalities, suggesting that the key impairment in SD is a breakdown in conceptual knowledge rather than a specific problem with language. Working memory and autobiographical memory, at least for the recent past, tend to be preserved. However, patients with SD perform poorly on standard anterograde verbal memory tests, such as word-list learning [28].

Behavioral symptoms may occur early or late in the clinical course. Patients with SD may present as less apathetic and more compulsive than those with FvFTD [27]. They may show interpersonal coldness and impairments in emotional processing. Patients with more marked right temporal lobe involvement tend to present with significant changes in personality, such as emotional disturbances, bizarre alterations in dress, and limited, fixed ideas [24].

Snowden et al., compared behavioral patterns and functional imaging in patients with FTD to those with SD. Whereas lack of emotional responsiveness was pervasive in FTD, it was often more selective in semantic dementia and particularly affected the capacity to show fear. Apathetic FTD patients also had a higher pain threshold, whereas patients with SD had an exaggerated response to pain. Overall, emotional, repetitive, and compulsive behaviors discriminated FTD from SD with an accuracy of 97% [12].

#### C. Progressive aphasia (PNFA)

Progressive non-fluent aphasia (PNFA) is a disorder predominantly of expressive language, in which severe problems in word retrieval occur in the context of preserved word comprehension. This disorder is associated with asymmetric atrophy of the left hemisphere [20]. Patients present with changes in fluency, pronunciation, or word finding difficulty. They do not present with behavioral problems until later in the disease [27]. In a study that assessed discourse in patients with both semantic dementia and PNFA, patients with PNFA had the sparsest output producing narratives and had the fewest words per minute [29].

### 5. Diagnosis

Patients with FTD should have a neuropsychiatric assessment, neuropsychological testing and neuroimaging studies to clarify the diagnosis. On neuropsychological testing, memory is relatively spared. Orientation and recall of recent personal events is good, but performance on anterograde memory tests can be variable. Patients tend to do poorly on recall-based tasks. A reduction in spontaneous conversation is common. Subjects also perform well on visuospatial tests, when the organizational aspects are minimized. The Folstein Mini Mental State Examination (MMSE) is unreliable for the detection and monitoring of patients with FTD, who frequently perform normally even when requiring nursing home care [26].

There have been several different classifications proposed to make the clinical diagnosis of FTD. The Lund and Manchester Group [30] initially established diagnostic criteria for FTD in 1994. Patients were required to present with at least two of the following signs or symptoms: loss of personal awareness, strange eating habits, perseveration or changes in mood. In addition, they had to have one of the following features: frontal executive dysfunction, reduced speech, or normal visuospatial ability.

Neary et al. [31] developed another set of diagnostic criteria, and specifically divided FTD into three prototypic syndromes. They have been delineated above as frontal-variant FTD, semantic dementia, and progressive non-fluent aphasia. McKhann et al., have sought to further define clinical criteria for FTD that can be easily used by clinicians in order to make a prompt diagnosis of FTD [13]. They proposed the following criteria for FTD:

1. The development of behavioral or cognitive deficits manifested by either

a. Early and progressive change in personality, characterized by difficulty in modulating behavior, often resulting in inappropriate responses or activities or

b. Early and progressive change in language, characterized by problems with expression of language or severe naming difficulty and problems with word meaning.

2. The deficits outlined in 1a or 1b cause significant impairment in social or occupational functioning and represent a significant decline from a previous level of functioning.

3. The course is characterized by a gradual onset and continuing decline in function.

4. The deficits outlined in 1a or 1b are not due to other nervous system conditions (e.g., cerebrovascular accident), systemic conditions or SA induced conditions.

5. The deficits do not occur exclusively during a delirium.

6. The disturbance is not better accounted for by another psychiatric diagnosis.

Neuroimaging can also be helpful in distinguishing FTD from other types of cognitive disorders. Typically, structural imaging shows anterior temporal and frontal atrophy, while functional imaging shows decreased perfusion of both frontal and temporal lobes [32]. MRI scans indicate that both PNFA and FvFTD show frontotemporal atrophy. The focus of the atrophy is in the left temporal lobe in PNFA patients and in both frontal lobes in FvFTD patients. In contrast, the mesial temporal lobes are atrophic in AD patients [33]. Imaging abnormalities in FvFTD usually appear later in the disease course. Tc-HMPAO-SPECT scanning can detect hypoperfusion in the ventromedial frontal region even before atrophy is evident. In the later stages of the disease, atrophy of the frontal and anterior temporal lobes becomes more apparent [26]. According to a recent report studying SPECT differences in FTD patients with different symptoms, patients with right frontal involvement meet the consensus criteria more frequently than patients with greater involvement in other areas.

Neurochemical changes in the behavioral presentation of FTD contrast with those of AD. There is evidence of less cholinergic deficit and more serotonergic disturbance in FTD than in AD. In fact, acetylcholinesterase and cholinergic acetyltransferase activities are well preserved in FTD. Serotonin dysfunction is linked with impulsivity, irritability, affective change, and changes in eating behavior. These are all common features of FTD. Additionally, the serotonergic system is associated with the frontal lobes, which are often affected in FTD. Using functional imaging, serotonin binding has been shown to be reduced in the frontal cortex in FTD. Thus, FTD has been described as mainly a post-synaptic pathology. Monoaminergic and dopaminergic alterations have also been reported in the literature [34].

### 6. Differential diagnosis

The differential diagnosis for FTD is broad. Some conditions that need to be considered in the differential diagnosis, include illnesses, that cause both cognitive and behavioral deficits, such as stroke, Parkinson's disease, Huntington's disease, hypothyroidism, HIV and substance abuse (primarily alcohol) [32]. FTD also overlaps with other neurodegenerative diseases, such as motor neuron disease, corticobasal degeneration and progressive supranuclear palsy [27].

FTD is most often mistaken for AD; indeed, many patients with pathologically confirmed FTD have been diagnosed with Alzheimer's disease during life. In a study by Miller et al., FTD was best differentiated from AD by using behavioral criteria such as early loss of social awareness, early loss of personal awareness, hyperorality, progressive loss of speech, and stereotyped and perseverative behaviors. Using these standards, the sensitivity for detecting FTD was 63.3% to 73.3% and specificity was 96.7% to 100% [35]. For a quantitative measure to distinguish between the two conditions, the Frontal Behavioral Inventory (FBI) has been developed by Kertesz et al. [36]. The FBI is a 24-item caregiver-based behavioral questionnaire designed for the diagnosis and quantification of FTD symptoms. Cognitive tests like the MMSE do not readily distinguish between FTD and AD. On the other hand, the FBI differentiated 98% of FTD and AD patients. The FBI tests areas such as apathy, aspontaneity, indifference, inflexibility, concreteness, personal neglect, disorganization, inattention, loss of insight, logopenia, verbal apraxia, perseveration, irritability, excessive jocularity, poor judgment, inappropriateness, impulsivity, restlessness, aggression, hyperorality, hypersexuality, utilization behavior, incontinence, and alien hand^5^. Another study reported that, compared to patients with AD, patients with FTD tended to develop symptoms at an earlier age, develop behavioral symptoms earlier in the course of their illness, and have less prominent memory loss. In addition, patients with FTD commonly present with motor abnormalities, which are not common in AD [32].

In 2002, Rascovsky et al. compared patterns of cognitive deficits between patients with autopsy-confirmed FTD and AD. They found that patients with FTD were more impaired than patients with AD on word-generation tasks such as letter fluency and category fluency. Conversely, patients with AD were more impaired than patients with FTD on memory tasks and visuospatial tasks such as block design and clock drawing [37]. Although similar findings have been reported in the literature [38-42] there is an overall lack of consistent findings in studies attempting to differentiate the cognitive profiles of FTD and AD. Possible reasons for the inconsistencies include improper matching of FTD and AD populations (i.e., comparing pa444etients at different stages of dementia); choice of neuropsychological test; and lack of pathologically-confirmed diagnoses [37].

### 7. Neuropathology

The typical changes seen in FTD are atrophy of the prefrontal and anterior temporal neocortex. Routine histology shows microvacuolation of the outer cortical laminae due to large neuronal cell loss, or transcortical gliosis [20]. Pathologically, FTD is heterogeneous; some cases may show tau- or ubiquitin- positive inclusions, or they may lack distinctive histological features [43]. Sensitive methods for detecting tau abnormalities and for ubiquitin are essential in the neuropathological evaluation of FTD [13].

Tau-protein is involved in the regulation of microtubule assembly and disassembly. For hereditary FTD, more than 50 different tau mutations have been identified in several families. Although the frequency of tau mutations in sporadic FTD is low, in patients with a family history of FTD the frequency of tau mutations ranges from 9.4 to 10.5%. These tau abnormalities may lead to aggregation or to disruption of microtubules, which in turn affects the intraneuronal transport system [44].

Kertesz et al. followed 60 patients who met criteria for behavioral variants of FTD to autopsy. They reported that the most common histological variety was motor neuron disease type inclusion, followed by corticobasal degeneration, Pick's disease, dementia lacking distinctive histopathology, and progressive supranuclear palsy. They also reported that tau-negative patients had an earlier age of onset [25].

Forman et al., studied whether specific clinical features in patients with FTD predict the underlying pathology in 90 patients with a pathological diagnosis of FTD. They reported that taupathies were more frequently associated with an extrapyramidal disorder, whereas patients with ubiquitin-positive inclusions were more likely to present with social and language dysfunction as well as with motor neuron disorder [45].

In 2001, an international group of scientists reassessed neuropathological criteria for the diagnosis of FTD. They recommended classifying neurodegenerative disorders associated with FTD into five distinct neuropathological categories, based on presence or absence of tau-positive and ubiquitin-positive inclusions, predominance of microtubule-binding repeats in insoluble tau, and presence of motor neuron disease-type inclusions. However, they emphasized that only probabilistic statements could be made when examining the causal relationship between neuropathological findings and clinical manifestations of neurodegenerative disorders, as it is unclear exactly how neurodegenerative diseases cause specific clinical syndromes [13].

### 8. Treatments

#### A. Non-pharmacological Treatments

The behavioral approach to treating FTD is a challenging matter for most clinicians. Livingston et al., conducted a systematic review of different psychological treatments available for the behavioral disturbances of dementia. Although this review was not specific to FTD and included studies on patients with other types of dementia, the results were noteworthy given the paucity of high-quality research in this area. They concluded that approaches with positive evidence to support them included techniques centered on individual patient behavior, and that psychoeducation intended to change caregiver's behavior could have an effect on the patient's neuropsychiatric symptoms lasting several months [46]. There is clearly a need for further studies to evaluate the efficacy of non-pharmacological approaches to the management of behavioral disturbances of dementia, particularly in patients with FTD.

In her review, Litvan., addressed the dilemma of caregiver burden in FTD. Although there have been no published studies of caregiver burden in FTD, she concluded the findings would likely be similar to studies with AD. Caregiver perception of burden is strongly correlated with distress and is related to earlier nursing home placement of patients. Caregiver distress is also associated with increased health care disturbances, needs, and costs for the provider. Caregiver burden is linked to decreased immunity and consequently increased vulnerability to infection. Social support has been shown to not only decrease caregiver stress but also to lower anxiety and promote better immune response. Therefore, it is important to provide support, education, and treatment to providers. Studies have suggested that early caregiver intervention may delay patient institutionalization and improve the quality of life for both the patient and caregiver [47].

Unfortunately, many FTD patients eventually require long-term placement. No standard method of structuring the transition to a long-term facility has been studied in this population. Such a change in environment may result in increasing disorganization, irritability, and agitation. However, patients with dementia do not uniformly respond to stress in the same way; hence, it may be best to consider an individualized approach to this transition. Patients at risk for elopement may require the use of a secure unit or "wanderguards." Patients with impairments in language may benefit from alternative means of communication, including careful attention to body language. In a few rare cases, patients have been transferred to secure units for medication management until stabilized [48].

#### B. Pharmacological Treatments

Table 2 summarizes the various studies of pharmacologic treatments in FTD. Selective Serotonin Reuptake Inhibitors (SSRIs) have been used with some degree of success in patients with FTD. Studies of these medications in this population have shown an effect on behavior but not on cognition [49]. Some studies have demonstrated deficiencies of serotonin in patients with FTD. Further, the anatomic and clinical presentation of this illness may correspond with serotonergic dysfunction (e.g. aggression and impulsiveness) [49]. Unfortunately, there have been few large-scale clinical trials published to date. Swartz et al., enrolled 11 patients with FTD in a three-month, open-label trial with the SSRIs sertraline, paroxetine, and fluoxetine. After 3 months of treatment, 9 of 11 subjects (82%) showed improvement in at least one of the following behavioral symptoms: disinhibition, depressive symptoms, carbohydrate craving, or compulsions. No subjects showed worsening of symptoms [50]. An open label, uncontrolled trial with paroxetine had good results as well. In this study, up to 20 mg/day was given to 8 patients with FTD. At 14 months, the subjects demonstrated an improvement in behavioral symptoms. This was coupled with decreased caregiver distress. Baseline scores of global performance, cognition, and planning remained stable, but there was a decrease in attention and abstract reasoning. Side effects were tolerable, and there were no dropouts [51]. Recently, a randomized placebo controlled trial with paroxetine was completed. Ten patients were given up to 40 mg/day and treatment assessments were done at the 6^th ^or 7^th ^week. Patients were found to have had no improvement on the Neuropsychiatric Inventory (NPI) or the Cambridge Behavioral Inventory (CBI). Furthermore, subjects actually demonstrated increased error rates on the reversal component of the visual discrimination task, the paired associates learning task, and delayed pattern recognition task. Interestingly, these three tasks have been shown previously to be sensitive to tryptophan depletion [52]. Ikeda et al., studied the reaction to fluvoxamine of 16 patients diagnosed with FTD in an open 12-week trial and reported a response in behavioral symptoms, especially stereotyped behaviors [53].

**Table 2:** Summary of various frontotemporal dementia treatment studies

**Treatment**	**Study Details**	**Outcome**	**Side Effects**
**SSRI's**	Swartz et al 1997: 11 patients; open-label (sertraline, paroxetine, fluoxetine)	9/11 had behavioral improvement	Diarrhea (1/11) Increased anxiety (1/11)
	Moretti et al 2003: 8 patients; open-label (paroxetine)	Behavioral improvement Reduced caregiver burden	Transient nausea (37.5%)
	Deakin et al 2004: 10 patients; RCT^a ^(paroxetine)	No improvement in NPI^b ^or CBI^c ^scores Impairment seen on learning tasks/recognition tasks	None reported
	Ikeda et al 2004: 16 patients; open-label (fluvoxamine)	Behavioral improvement Decreased stereotypes	None reported
**Trazodone**	Lebert et al 1999: 14 patients; open-label	Improvement in delusions, irritability, aggression, disinhibition (dose-dependent)	None reported
	Lebert et al 2004: 26 patients; randomized controlled trial	Improvement in irritability, agitation, depression, eating disorders	None reported
	Lebert et al 2006: 26 patients; open-label extension of 2004 RCT	Improved behavioral symptoms; improved NPI score	Hypotension (15%)
**Antipsychotics**	Curtis et al 2000: 1 patient; case report (risperidone)	Improved psychosis and social interactions	Akathisia Mild parkinsonism
	Pijnenburg et al 2003: 24 patients; retrospective chart review (majority of patients given typical antipsychotics)	Not reported	Extrapyramidal symptoms (33%) Sedation (12.5%)
**Others**	Moretti et al 2004: 20 patients; open-label (rivastigmine)	Improved behavioral symptoms Reduced caregiver burden No change in MMSE^d ^score	Nausea (25%) Muscle cramps (20%) Blood pressure changes (15%)
	Goforth et al 2004: 1 patient; case report (methylphenidate)	Improved behavioral symptoms	None reported

^a ^= randomized controlled trial; ^b ^= Neuropsychiatric Inventory; ^c ^= Cambridge Behavioral Inventory; ^d ^= Mini-Mental Status Exam

Lebert and Pasquier, evaluated 14 subjects with FTD treated with trazodone, an atypical serotonergic agent. Trazodone's activity is mainly based on post-synaptic 5-HTa/2c antagonist effects. It has a 5-HT1a agonist effect by its metabolite and has modest selective serotonin reuptake inhibitor effects. Essentially, trazodone increases extracellular 5-HT levels in the frontal cortex. In the trial, the patients were treated for 6 weeks. They received 150 mg/day for the first 4 weeks and 300 mg/day during the final 2 weeks. All of the patients showed a dose-dependent improvement in behavioral symptoms. After 4 weeks, they exhibited decreased delusions, aggression, anxiety, and irritability. Six weeks of treatment resulted in additional decreases in depression, disinhibition, and aberrant motor behavior [54]. A randomized, double-blind, placebo-controlled cross-over study with trazodone in FTD was completed in 2004. Twenty-six patients were evaluated using the NPI. A significant decrease (p = 0.028) of more than 50% in the NPI score was observed in 10 of the patients on trazodone. Overall, a decrease of over 25% in the total score for behavioral disturbances was seen in 61% of the FTD patients. The improvement was mainly seen in irritability, agitation, depressive symptoms, and eating disorders. Trazodone was generally well tolerated [34]. These same authors completed an open-label extension of the trazodone trial for two years following the end of the double-blind trial. They reported improved behavioral symptoms and significantly (p = 0.028) improved score on the NPI. Cognition was less impacted, as 9/16 patients had a decrease in MMSE score of greater than three points, while 7/16 patients had either no change or only minor decrease in MMSE score. Hypotension was reported as the single adverse effect (15% patients) [55].

Dopamine use for the treatment of FTD is controversial. In practice, behavioral disturbances are occasionally managed by D2 blockers, but it is possible that patients may benefit more from treatment with selective dopamine agonists. Recent studies have proposed that bromocriptine, a D1 and D2 dopaminergic agonist, may improve selective frontal features. An open label study suggested that bromocriptine improved perseveration in dementia [56]. A case report using methylphenidate and quantitative EEG correlated with SPECT demonstrated that profound left greater than right bi-frontotemporal slowing partially normalized after methylphenidate administration. This finding occurred in the context of significant behavioral improvement in the patient [57].

The use of neuroleptics for the treatment of agitation in dementia is controversial. The FDA recently determined that the use of atypical antipsychotics for the treatment of behavioral disorders in elderly patients with dementia is associated with a higher mortality than treatment with placebo, specifically due to cardiac-related events and infections [48]. One case reported an improvement in psychotic symptoms and social interactions in a 42 -year old woman with Pick's disease treated with risperidone [58]. Some authors maintain that patients with FTD are especially sensitive to extrapyramidal side effects of neuroleptics. Pijnenburg et al studied the appearance of extrapyramidal side effects in 100 patients with FTD and found that around 33% of patients treated with neuroleptics developed these side effects. They also reported that in some instances it took several weeks for the EPS to resolve [59]. The safety and efficacy of antipsychotics in FTD needs to be thoroughly studied in order to establish if they are of value in the treatment of these patients.

Regarding cognitive enhancers, Moretti et al., studied the effect of rivastigmine, an acetylcholinesterase and bytyrylcholinesterase inhibitor, in 20 patients with FTD for 12 months. They found a general amelioration of behavioral changes and reduced caregiver burden, although they did not find any differences in the progression of cognitive impairment as measured by the MMSE [60].

Pathological tau proteins are biochemical markers found in various degenerative dementias, including several subtypes of FTD. Tau mutations, though, have only been discovered in autosomal dominant FTDP-17. Novel therapeutics will likely focus on targets linked to disease pathogenesis, which are likely to be different for each FTD subtype. Agents that prevent the expression or accumulation of tau represent a future direction of therapy. So far, lithium has been shown to decrease tau phosphorylation and aggregation in transgenic mice [61], but lithium is generally poorly tolerated in the elderly.

## 9. Conclusion

FTD is a common and severe neurodegenerative disorder, which has drawn a lot of attention among the medical community in the last decade. FTD is estimated to account for 20% of cases of degenerative dementia with presenile onset [12], and post-mortem investigations have reported a relative frequency of FTD of 3–10% [44]. Its pathophysiology is still unclear, and further research should be directed at understanding and developing new diagnostic and therapeutic modalities to improve patients' prognosis and quality of life.

## Competing interests

The author(s) declare that they have no competing interests.

## Authors' contributions

NDW and RT wrote the main document. RA and KW contributed with specific areas of the document and with the tables. All authors read and approved the final manuscript.
